# Bad Blood or My Blood: A Qualitative Study into the Dimensions of Interventions for Mothers with Children Born of Sexual Violence

**DOI:** 10.3390/ijerph16234810

**Published:** 2019-11-29

**Authors:** Elisa van Ee, Jorin Blokland

**Affiliations:** 1 Psychotraumacentrum Zuid-Nederland of Reinier van Arkel, Bethaniestraat 10, 5211 LJ ‘s Hertogenbosch, The Netherlands; 2 Behavioral Science Institute of Radboud University Nijmegen, Montessorilaan 3, 6525 HR Nijmegen, The Netherlands

**Keywords:** sexual violence, rape born, mother–child relationship, stigma, attachment, trauma

## Abstract

Research has shown that there is a negative relation between the experience of sexual violence of mothers and the well-being of their children. When a child is born out of sexual violence, the origin of the child is connected to the traumatic experience. Despite the difficult maternal task of navigating this relationship, research on interventions for mothers with a child born of sexual violence is absent. The current qualitative study was designed to gather expert knowledge of twelve clinicians on the dimensions of interventions for these mothers and their children as a first step in the development of good clinical practice for interventions. Using thematic analysis, the interviews were transcribed, coded and analyzed. Three building blocks for interventions for mothers and their children born of sexual violence were identified: building a secure attachment, reduction of trauma-related symptomatology, and addressing stigmatization. Clinicians describe many factors that need to be taken into account in treatment but emphasize the importance of the therapeutic relationship to be efficacious. The foundation of a strong therapeutic relationship together with the building blocks are the elements for good clinical practice on interventions for mothers with a child born of sexual violence.

## 1. Introduction

Sexual violence, defined as any act of a sexual nature that is committed without consent, can have a profound impact on the survivor, the community and even the next generation. Research has shown that there is a negative relation between the experience of sexual violence of mothers and the well-being of their children [1,2,3,4]. This relation is mediated by mental health problems of the mother, such as symptoms of depression and post-traumatic stress [5,6,7]. Maternal symptoms of post-traumatic stress and depression may affect the mother–child interaction and thereby child development. For example, traumatized mothers can be less sensitive, available, involved and more intrusive and hostile in their interaction with their children [8,9]. These affected interactions raise concerns for the formation of a secure attachment relationship. Indeed, a relation between parental symptoms of post-traumatic stress and insecure or disorganized attachment styles of their children has been found [10,11]. Similar results have been found for depressed mothers and the relationship with their children [12,13,14]. 

Considering these results and the nature of the traumatic experience, it has been suggested that relational models of care are most appropriate for mothers with a history of sexual violence [8]. Using a relational model, therapeutic interventions encompass a focus on alleviation of mental health disturbances of the mother and on restoration of relationships. Indeed, mental health is more complex than a lack of symptomatology and is indispensable to personal well-being, interpersonal relationships and family functioning [15]. In such an approach application of evidence-based quality standards to reduce symptomatology [16,17] go hand-in-hand with interventions to improve relational functioning. The fostering of a secure attachment relationship between mother and child with (early) childhood programs that are informed by principles of attachment theory might be particularly effective for children of at-risk-mothers [3,6,8].

The prevalence of children conceived in the act of sexual violence is unknown. Global estimates indicate that 30% of women report some form of physical or sexual violence by their intimate partner in their lifetime, but the number of pregnancies resulting from violence in intimate relationships remains unknown [18]. In times of complex emergencies, like war, gender-based violence strongly increases. In different settings and populations, reports show high numbers of sexual violence toward women and girls [19]. In the Democratic Republic of Congo, the prevalence of sexual violence has been estimated to be 12% among women, and the prevalence of sexual violence-related pregnancies to be between 6% and 17% among sexual violence survivors [20,21]. In Nigeria, Boko Haram released 82 Chibok schoolgirls. Many of these girls reported being sexually abused, and some of them came back pregnant or with children referred to as “bad blood” [22]. In these cases, the mother has to deal with the physical health and mental health consequences of sexual violence while at the same time navigating the relationship with their child born of sexual violence. 

When a child is born out of the experience of sexual violence, the origin of the child is connected to the traumatic experience. In these cases, clinical case reports describe motherly feelings of rejection and hatred because the child is a constant reminder of the sexual violence oscillating with feelings of motherly love because the child is innocent and of their own [23,24]. Even if the child never meets the father, to the mother, the children’s identity can be linked to that of their rapist fathers [23,25]. In particular, this ambivalence, when prevalent in the mother–child relationship, is a risk-factor for the formation of a secure attachment relationship [9,11,26]. In addition, the experience of sexual violence, even more so when sexual violence leads to pregnancies, is a risk factor for rejection and social isolation [21,27,28]. The reaction of the community becomes a culture medium for chronic stress and the suffering of mother and child. Due to the intensity of feelings and reactions, some mothers choose to abort, reject or abandon the child [29,30]. Physical abuse and infanticide by both mother and community members has been documented [31]. Nevertheless, some mothers decide to raise their child born of sexual violence. These mothers face “the difficult task of differentiating the baby from the experience” [32] (p. 128). 

Despite this difficult task, research on interventions for mothers with a child born of sexual violence is, to our knowledge, absent. Even consensus on good practice addressing the specific difficulties of mothers and their children born of sexual violence is unavailable. Therefore, the aim of the current qualitative study was to gather expert knowledge on the dimensions of interventions for these mothers and their children and thereby to contribute to a first step in the development of good clinical practice in the treatment of this population. 

## 2. Method

### 2.1. Participants

Thirteen clinicians (psychiatrists, psychotherapists, clinical psychologists and psychiatric nurses), male (*n* = 1) and female (*n* = 12) and from diverse countries (Bosnia (*n* = 2), Lebanon (*n* = 2), the United States (*n* = 3) and The Netherlands (*n* = 6) were interviewed. The selection criterion for interviewees was a minimum of two years of experience in offering supportive interventions to mothers who experienced sexual violence and were raising a child born as a result. The expertise of the interviewee had to be confirmed by at least one other clinician. On average, the interviewees had 10.4 years of experience (range 2–25 years); five worked with victims of torture and war violence, mostly refugees or victims of human trafficking, three Dutch clinicians worked with Dutch inhabitants and foreign clients who have had experience of rape, one clinician worked with women who experienced sexual violence in military service, one clinician worked in post-war environments, and two clinicians from the US had worked both in their own country and abroad in post-war environments with traumatized women and children. The interviewed clinicians worked with mothers who experienced sexual violence by family members, partners, team members during military service, strangers in the street, war time, or sex trafficking. One clinician with insufficient counselling experience with the target group was excluded from the analyses, which resulted in a final sample of 12 complete interviews. All subjects gave their informed consent for inclusion before they participated in the study. The study was conducted in accordance with the Declaration of Helsinki, and the protocol was approved by the Ethics Committee of Reinier van Arkel.

### 2.2. Procedure

Experts working with children born of sexual violence were identified via organizations and networks working with this population. They were invited by email to take part in the study. The scope of the study was explained, and the clinicians were asked to respond if they wanted to participate. In addition, they were asked to identify potential respondents working with this target group (i.e., ‘snowball sampling’). A meeting via Skype or in person was scheduled—in which, informed consent was confirmed. The interview took approximately one hour. 

Based on a literature review on the needs of mothers with a child born of sexual violence [23], an interview protocol was developed by a panel of researchers and experienced clinicians and conducted by trained social scientists. The interview contained open-ended questions aimed at identifying the mental health needs of mothers and children, content of interventions, efficacy of interventions, and gaps in care. Examples of questions are: “What kind of interventions do you use?”; “In your opinion, what makes these interventions particularly useful in the treatment of mothers and their children born of sexual violence?”; “Which moments in therapy are moments of change for these women? And for children?”; “What kind of knowledge do you miss when you treat these women and children?”.

All interviews were conducted in Dutch or English, voice recorded, and transcribed. To explore the recurrent themes in the data, thematic content analysis was used [33]. The coding process involved three levels of data coding, starting with line-by-line underlining key words and phrases, followed by summarizing key phrases into codes and organizing the data thematically. In a process of analytic triangulation, two people analyzed the data. Differences were discussed within the research team until consensus was reached. Open, axial, and selective coding were used to illuminate categories. In the final stage, we organized the data in terms of the dimensions of interventions. Good interrater reliability (kappa = 0.737) was established for the codes on four interviews (33%).

## 3. Results

Multiple codes emerged from the data through the analysis and were grouped into three major themes related to the dimensions of interventions. These three basic themes are: 1. process of treatment, 2. aims of treatment and 3. efficacy of treatment. 

### 3.1. Process of Treatment

#### 3.1.1. Start of Treatment

When mothers seek help, most often it is a request for support not related to the sexual violence or the origins of the child. Reasons to apply for help are behavior problems of their children, contact with child protective services, financial problems, difficulties with impulse regulation, difficulties in relationships, and the presence of psychiatric problems.

*“I have never seen an application for treatment which stated there is a child born of sexual violence and the mother has problems with that. Can you help? I have never seen this. The application states the child has emotional or behavioral problems or a mother is in treatment and during treatment they discover she has a difficult relationship with her child and when they explore this further it comes out.”*

When the mother opens up about the origins of the child, soon enough, the question of how to take care of the child and how to become more attached to the child comes to the front:

*“How can I be a mother for this child, what can I do to accept my child?”*

*“They need the basic support how to take care of their child and how to see the needs of the child.”*

#### 3.1.2. Modality of Treatment

If available, therapy consists of different modalities: individual, system, and group therapy. Individual therapy can help mothers to process their traumatic experiences and to live in the here and now. Group therapy usually has the form of psychoeducation and exercises with mother and child, with the focus on attachment. Some clinicians work with the mother and child together, especially when the children are young (*n* = 7). Others focus more on the mother or the child. The importance of working with the whole system, however, was emphasized throughout the interviews:

“[…] so as I was teaching the coping strategies to the child, and the parent would be in the room learning them as well. […] I found it most optimal when we could work with the family system, and then also have an individual component for the parent.”

Another form of group therapy can be aimed at the mothers, with the focus on empowerment achieved by doing something useful together and building a social network. 

#### 3.1.3. Important Themes

Three themes that need to be addressed in therapy are: stigma, the father of the child, and the choice of keeping the child. In some post-war countries, stigma has been introduced as a topic in UN programs to decrease the stigma toward women who have been sexually abused. For clinicians, it remains a challenge to break through this prevalent feeling of stigma and shame. This is an important topic to address in treatment because clinicians acknowledge that it supports women when they perceive rape as a violation of human rights and not their individual shame. One clinician mentioned that feelings of guilt and shame causes many dropouts from therapy, which makes it important to address in therapy. Secondly, mothers need to be prepared for questions from the child regarding their father. Often, mothers are afraid of the reaction of their children. It is mentioned multiple times that they can hide their secret for years.

*“Some of these mothers hide that their children born out of rape and say to them, for example, that their father was a war hero or that he was killed during the war. And some of the children actually guess that they are born out of rape, but no one wants to tell them the truth.”*

A mother needs to decide whether she prefers to proactively address the origins of the child or prepare herself for the moment the child starts questioning. Finally, clinicians spoke about the need, even when challenging or uncomfortable to the clinician, to address the pregnancy and the choices that were made, for example to keep the baby.

*“[…] you need to address this fear. Or if you don’t do that, you’re actually placing the responsibility on them.”*

Adoption, especially when recovery fails to happen in the relationship, needs to be addressed. 

### 3.2. Aims of Treatment

Three aims of treatment were identified by clinicians: (1) building a secure attachment between mother and child, (2) the reduction of post-traumatic stress and trauma-related symptomatology, and (3) addressing stigmatization.

#### 3.2.1. Building a Secure Attachment between Mother and Child

All clinicians discussed the challenge of overcoming the ambivalence of the mother towards the child and perceive this problem as very distinct from other traumatized mothers and their children:

*“These mothers do not know how to perceive their child and how to perceive themselves. They see their child as dirty or bad and themselves as damaged or dirty or someone you have to be ashamed of. And at the same time, they have to shape their motherhood.”*

*“[…] the biggest problem is this ambivalence, love and hate and feelings of guilt at the same time. They feel love for the children and then they hate, they say they remind them of the trauma and therefore of their life.”*

Some mothers cannot stand certain behaviors of the child that remind them of the father. An extreme fear that the child will look like him, especially if the child is a boy, can dominate the relationship.

*“These mothers react different to boys because they remind them of their father. And when they grow older it becomes more difficult to handle them and to touch them.”*

According to the interviewed clinicians, the perception of the child becomes clouded. They describe how mothers tend to overestimate the hostility of the child (“they don’t want to listen”), but also their ability to understand (“they need to know this”). In some cases, the ambivalence can lead to unpredictable, sometimes even violent, parenting.

*“They have really a hard time connecting to the child. For example, in the delivery room they don’t want to hold their baby, they don’t like their baby, they want to get rid of their baby. (…) One mother actually tried to kill her child, she put a pillow over his mouth to stop the crying. So definitely not each of the mothers were that extreme, but you know, I worry about child abuse, I worry about neglect, (…) And I think it [abuse or neglect] centers around disconnection and depression.”*

*“One mother with a child born of sexual violence said for example: “I can’t do that because I am stressed [because of the child].” And I asked her: “What do you do then?” And she said: “I hit my child.” And I asked her: “How long do you hit him?” And she said: “I hit him until I feel the pain in my hands.” Then you know that she is not able to empathize with the child. And I continue to ask questions about what she thinks the child needs and I ask her if she was a child of two years old what she would need instead off… and we role play… all to enable the mother to think this child needs me and the needs of my child are this and that.”*

*“And why do you think this leads to change?”*

*“Because the child is seen as a child with needs and not as a child of the enemy that irritates or triggers the mother.”*

Some clinicians (*n* = 5) mentioned that in particular during dissociation, unconscious and non-intentional distancing of one’s own bodily sensations or the environment [34], the use of violence can be profound, while other interviewees (*n* = 3) notice that the presence of the child can prevent the mother from dissociating, which is why they carry their child with them all the time. These mothers tend to see the child as their belonging, their gift, while at times they become tired of caring and reject. 

In all interviews, the challenging attachment bond between mother and child was the most important topic.

*“I honestly think that the attachment needs are the greatest.”*

To support a secure attachment between mother and child, emotion regulation and mentalization, the capacity to perceive and understand mental states of the self and the child that help to explain and predict feelings, thoughts and behavior [35], is enhanced. Methods or techniques are used such as relaxation and coping skills, wait-watch-and-wonder, play therapy, mindfulness, window of tolerance exercises, parent–child interaction psychotherapy, video feedback, and behavioral parenting strategies.

*“Video feedback works really well (…) because they can see what they do well, little positive moments or moments of enjoyment. Sometimes those moments are even not there and we show them the moment that they sit a little closer or the moment a mother looks at her child.”*

By observing the behavior of the child in different contexts and monitoring the development of the child, clinicians help mothers to identify the needs of the child and to respond to those needs.

*“You continuously switch between three levels; you talk about how the mother experiences her child and herself as mother, and how that affects the child and the relationship, in the present and what she would like for the future.”*

*“It helps to differentiate between how the child was conceived and the child itself because on one hand you can’t separate it completely but on the other hand it shouldn’t be projected on the child.”*

#### 3.2.2. Reduction of Post-Traumatic Stress and Trauma-Related Symptomatology

All interviewees mentioned that most of these mothers have mental disorders as a result of the experience of sexual violence, such as post-traumatic stress disorder (with dissociation), anxiety, depression (whether or not postpartum), self-injury, substance abuse, low self-esteem, identity problems, personality disorders, and prolonged grief. It is not uncommon that the experience of sexual violence adds to pre-existing life events for example childhood sexual trauma, childhood neglect, unsafe relationships or pre-existing psychiatric disorders. However, compared to other populations, the intrusiveness of the sexual trauma is a symptom that stands out, as a child is born out of this experience. The child constantly reminds the mother of the experience and represents trauma to them.

*“When you keep your child from sexual assault you are getting a dose of trauma every day you know”*

The behavior of the child triggers memories from the sexual violence, which can result in flashbacks or dissociations, fueling a downward spiral of fear, negative perceptions, and more triggers.

*“So if the child doesn’t listen […] the mother thinks you are just like your father who just did what he wanted and did not listen. To them it is a one on one repeat of their trauma.”*

All clinicians, therefore, emphasize the importance of trauma focused-therapy combined with psychoeducation and attachment work. Some specific trauma-focused interventions were mentioned: Trauma-Focused Cognitive Behavioral Therapy, Imaginaire Exposure Therapy, Narrative Exposure Therapy, Sensori Motor Therapy, and Eye Movement Desensitization and Reprocessing. The importance of attending to feelings of shame and guilt during trauma therapy was highlighted—feelings of shame and guilt not only toward themselves but also toward their children. On the one hand, mothers perceive themselves as dirty and violated; on the other hand, it is not uncommon that these women feel guilty for not being the mother they would like to be. Most clinicians, therefore, speak about a mix in treatment, as one clinician explained:

“[…] so the exposure therapy plus the attachment work plus the meaning making. […] The science is the exposure therapy, the coping skills, the psychoeducation… but the art is the knowing what to attend to at what time.”

#### 3.2.3. Addressing Stigmatization

In most countries, it is taboo to talk about the topic of sexual violence and especially the bearing and raising of a child born of violence. Therefore, mothers remain silent due to the fear of rejection.

*“Because I could not believe that even after eighteen years they are coming to therapy and are hiding this.”*

Clinicians emphasize how they view sexual trauma as inflicted by the individual and perpetuated by the collective. One key component in therapy, therefore, is to make these women a part of society again and to help them build a strong network with friends and family.

*“One thing I focus on in therapy is the community. What I always try is to increase resources for the mother and child.”*

*“It is very important to work with the family, to explain them about the causes and the consequences to resolve this issue of stigma and shame… and supporting them to understand that the child is not guilty and that they should also support her in her need and vision to bring the child home.”*

Isolated mothers from collective cultures tend to miss their extended family—others who help with caring for the children and who are involved in everyday struggles. They long to feel accepted and supported by a group they are part of. One possibility to achieve this is group therapy in which mothers share their experiences and learn from each other’s feedback. Another possibility is group work aimed at social support in their own environment. These kinds of interventions are directed at rebuilding a social fabric, rebuilding relationships with the family who sometimes rejected the mother, education, or empowerment (e.g., handcraft projects). To truly reduce stigmatization, however, clinicians remarked that educating society on sexual trauma and the role of stigma is needed. However, the means to address stigmatization adequately are lacking.

*“It’s important to show them that it’s not only them, it’s not only their psychological symptoms that are happening. It’s also the responsibility of the community and you have to say it when it’s unfair, tell them that it’s so unfair, this is not right, and you did nothing wrong to deserve this and they have to hear it multiple times and internalize it.”*

While all clinicians subscribe to the importance of both trauma, attachment and community work, it is of interest that clinicians working in high-resource countries emphasize attachment and trauma work, while clinicians working in low-resource countries emphasize community work.

### 3.3. Efficacy of Treatment

As a clinician, what needs to change to know that the intervention is efficacious? Clinicians define two levels of moments of change in treatment. The first level is the self and what one has been through. When mother and child, for example, find rest in their traumatic experiences and start to have fun together, or when the meaning of the traumatic experience changes for the mother and she starts to feel in control again. The second level is the acceptance of the child and being able to differentiate the child from the trauma. A moment of change between mother and child, for example, is when the mother realizes that the child is not a copy of the father, that the child is part of her, and that the mother will open herself up for the child’s identity. The consequence of such a change is that mothers can focus on the parts of the child that they are proud of, be more sensitive, maintain eye contact, reduce hostility, and respond. Children can start to feel competent, accepted, and loved. It is the start of building a secure relationship.

Whereas some mentioned that current treatments are efficacious (*n* = 6), others mentioned that a lack of knowledge (*n* = 7) and a lack of effective treatments (*n* = 4) for this specific population is a challenge:

*“[…] I don’t think we have a full understanding of what is going on inside them.”*

*“[…] If the question is, ‘are there any psychological theories that fully understand the nature of becoming pregnant from rape,’, I would say no, there is none.”*

*“I have the opinion that the mothers and children do not receive the treatment they need.”*

To become efficacious in therapy, these clinicians foster a relationship of trust (*n* = 6) as the foundation of their work. Clinicians highlight the importance of being authentic and transparent as a clinician and, at the same time, alert and sensitive. In order to build trust, it is important to acknowledge the request for help from the mother and adapt to the themes that are important to her while avoiding triggers at the start of therapy.

*“If the mother is seen and heard in her needs and if she feels safe enough to share, it enables her to see her child and offer the child safety. Actually, I think it is sort of a parallel process.”*

According to these clinicians, it is the responsibility of the clinician to create a safe environment in which mothers can share their deepest emotions including those that are difficult to share, such as ambivalence toward the child, to create an environment where mothers can feel understood.

*“You have to listen to the mothers and just let them tell and accept them, even as the mother says: “I can’t look at this child” or “I wish he was never born” or “I don’t want this child, she drives me crazy”. You have to accept those thoughts as a human reaction.”*

Timing is essential, and clinicians agree that mothers should be given time to speak about their trauma and feel respected when they are not able to share their experiences yet.

*“My advice is to take time.”*

In timing interventions, clinicians describe how they need to come to an understanding whether delaying trauma work is avoidance or whether the mother is not ready to cope. In their opinion, the mother sets the pace, not the therapist. Therefore, the mother decides when she is ready.

*“We have to make sure the person in the room feels safe and realizes that the event is over and push them a little. Some of these women are still raising their children so the trauma is still kind of there, so figuring that out, but more than the cognitive safety of it, it is the psychological safety.”*

Some clinicians (*n* = 3) recommend psychodynamic work to understand the patient’s patterns. Equally important to them is to understand the therapist’s patterns—for example, the therapist’s assumptions regarding sexuality and the innocence of a child, which can lead to avoidance by the therapist or the mother.

It is of interested that the need to base treatment on the individual situation (*n* = 7) is most often mentioned. Every case is unique, and treatment needs to be adapted accordingly with a focus on their perceptions of themselves, their child, and the world.

*“I think there should be something that is coming from learning from the population itself. Not bringing a new theory and applying it on the patients. Make it inspired from the patients. […] We should have a new intervention because it’s a very unique population. But no, because we will be falling again in the same problem which is standardized stuff. The more we make things manualized, the more likely we lose contact with the person. […] It’s when you instead of developing or learning techniques that you can apply with this population, how about you get to know more about the stories and the background and the culture of these people and how their mind frame changes and to learn to use their language. […] And this way in therapy, you can have like not an observer from outside, no. You’re within the patient’s mental set.”*

## 4. Discussion

The aim of this study was to gather expert knowledge on the dimensions of interventions for the mothers and their children born of sexual violence as a first step in developing a framework for good clinical practice. The clinicians shared valuable insights into the specific and complex issues involved in treatment for this target population and identified three aims for treatment: building a secure attachment between mother and child, reduction of post-traumatic stress symptoms and trauma-related symptomatology, and addressing stigmatization. Interventions are preferably delivered in different modalities, namely individual, system, and group interventions. While clinicians agree on these three aims, it is of interest that clinicians working in high-resource countries emphasize attachment and trauma work, while clinicians working in low-resource countries emphasize community work. An explanation for this difference might be embedded in the context of the work of these clinicians. A lack of clinicians, specialized care, or services in general is omnipresent in post-conflict settings and clinicians report that they “have to be creative in solutions” to finance specialized care. A lack of resources might force them to prioritize their aims in treatment differently. It is of note that clinicians working in high-resource countries call attention to the risk of too much specialization and the need to change the mental health system (*n* = 4). Separate departments for children and adults, or separate care programs for disorders prevents the needed integration of care for mother and child. In addition, a knowledge gap in addressing stigmatization can be noted, according to these clinicians, the means to address stigmatization adequately are lacking. These findings are in line with the results of a Delphi study on priorities in care for mothers with children born of sexual violence—in which, experts deemed community work on stigmatization highly relevant, but not very feasible [36]. The attention given in low-resource countries to the response of the collective is commendable, but to support the development of effective integrated care and social support, more attention of funders, researchers and clinicians is needed for community-based work as part of a relational perspective on mental health.

**Figure 1:**
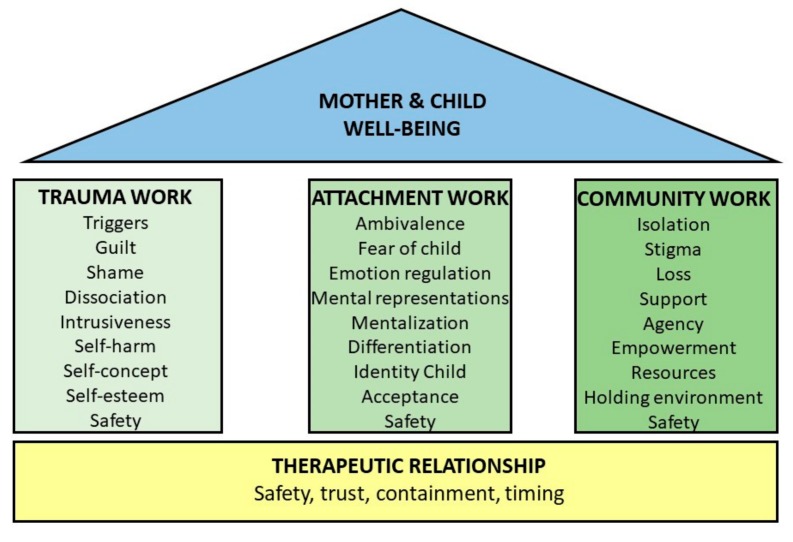
Building blocks and themes for interventions for mothers with children born of sexual violence.

Despite the range of contexts in which these clinicians work with mothers and their children born of sexual violence, all clinicians underline the importance of three building blocks for interventions in which certain themes are addressed (see Figure 1). Clinicians feel confident to a certain extent that by using these building blocks, they can deliver an efficacious treatment to these mothers and children. Synthesizing their responses, efficacy seems to be determined by the ability of a clinician to establish a safe therapeutic relationship with the mother and the child. A relationship built on trust and security, an investment in the person within a specific context, an ability to time and take time, and an understanding of the other and the self within the therapeutic space are indispensable ingredients. In this therapeutic relationship, meaning is co-created “because you circle gradually closer and closer until you end up at something that is understood by both the therapist and the client” [37] (p. 40). It is a therapeutic relationship in which besides post-traumatic stress disorder issues of loss and injustice, meaning and identity become meaningful as these issues can be of greater concern for the traumatized mothers and for their children [38]. Research has shown the contribution of a therapeutic relationship in effective therapies [39,40,41] and clinicians illuminate how, using the building blocks, the therapeutic relationship is their foundation to enable the application of tailor-made evidence-based interventions. In this difficult to reach population with complex needs, a safe therapeutic relationship contributes not only to the efficacy of interventions but is also the cornerstone that makes interventions possible. Interventions aimed at fostering a secure attachment, reducing trauma-related symptomatology and addressing stigmatization, build on the foundation of a strong therapeutic relationship are the elements for good clinical practice on interventions for mothers with a child born of sexual violence.

The current study was subject to limitations. Interviewing therapists instead of the mothers is a prominent limitation. The presented results, therefore, can only be seen as perceptions and interpretations of clinicians. The number of interviewed clinicians was limited; they worked with a variety of populations with different types of sexual violence and half of them were located in Western countries. It is of interest though that clinicians’ responses were in sync while working within diverse populations from different cultural backgrounds, which makes the information more representative for treatment within different settings and among different populations. As this study aimed to gather knowledge on good clinical practice in the treatment of mothers and children born of sexual violence, future research could focus on the perception of mothers and children on effective interventions and the development and validation of a specific treatment for this target population while taking into account the key points that are mentioned by the clinicians.

## 5. Conclusions

In conclusion, the current study identified three aims of interventions for mothers and their children born of sexual violence: building a secure attachment between mother and child, reduction of post-traumatic stress symptoms and trauma-related symptomatology, and addressing stigmatization. Interventions preferably consist of different modalities—individual, system, and group therapy. Stigma, the father of the child, and the choice to keep the child are themes that need to be addressed at some stage in treatment. Clinicians described many factors that need to be taken into account in treatment but emphasized the importance of the therapeutic relationship to be efficacious. It is the cornerstone for the three identified building blocks in interventions (see Figure 1). Despite the complexity of difficulties within this hard-to-reach population, there is hope that mothers and children can recover from this trauma that defines their relationship. As a mother said: “Now I realize he is half my own blood, and he can’t do anything about all of this.”
